# Advancing global pediatric cancer survivorship care: barriers, facilitators, and implementation strategies

**DOI:** 10.1007/s11764-025-01958-3

**Published:** 2026-01-19

**Authors:** Nicholas B. George, Sara Malone, Amela Sijecic, Grace Cross, Melissa Martos, David Noyd, Lisa M. Force, Nickhill Bhakta, Matthew J. Ehrhardt, Maura M. Kepper

**Affiliations:** 1 University of Washington, Seattle, WA USA; 2 Washington University in St. Louis, St. Louis, MO USA; 3 St. Jude Children’s Research Hospital, Memphis, TN USA

**Keywords:** Survivorship, Global oncology, Implementation science, Barriers and facilitators, CFIR, ERIC

## Abstract

**Background:**

As more children with cancer experience improved treatment outcomes across the world, challenges arise with ongoing care needs due to a higher risk of mortality and chronic health conditions compared to the general population. Addressing global pediatric cancer survivorship care, especially in low- and middle-income countries (LMICs), is of growing importance. The current study used the Consolidated Framework for Implementation Research (CFIR) and the Expert Recommendations for Implementing Change (ERIC) to examine the barriers, facilitators, and strategies for implementing childhood cancer survivorship care in LMICs.

**Methods:**

Using a larger review (PROSPERO registration CRD42021242548), a total of 8,456 articles were considered. The search identified nine eligible articles mentioning determinants of survivorship care across LMICs. Data were extracted from these articles using the CFIR domains of outer setting, inner setting, and individuals. Assigned CFIR constructs were then paired with ERIC strategies using the CFIR–ERIC matching tool.

**Results:**

The nine studies were published between 2003 and 2020, representing the following countries: India, Brazil, Turkey, China, and Thailand. Inner setting barriers included lack of available resources—funding, space, materials, and guidelines. Outer setting barriers were related to financing, policies, and laws. Individual patient barriers reported were low health literacy, distance to care centers, and low prioritization of follow-up. No common facilitators were noted. The most feasible ERIC strategies for the barriers identified were accessing new funding, developing and distributing educational materials, developing resource-sharing agreements, and conducting local survivorship care needs assessments.

**Conclusions:**

There are limited existing data evaluating childhood cancer survivorship care barriers in LMICs. Of the studies we identified, low resource availability was a frequently reported barrier. As accessing funding can be difficult, resource sharing of contextually adapted guidelines and educational materials can serve as an implementation strategy to improve survivorship care globally.

**Implications for Cancer Survivors:**

Enhancement of research efforts in resource-limited settings will address key knowledge gaps critical for implementation of global survivorship care and equity throughout the cancer journey.

## Background

Approximately 400,000 children worldwide are diagnosed with cancer annually, disproportionately affecting children in low-and middle-income countries (LMICs) [1, 2]. In recent decades, cancer treatment and supportive care advancements have yielded steady improvement in outcomes for children with cancer. More than 85% of children diagnosed with cancer in high-income countries (HICs) will be cured and experience extended survival into adulthood, resulting in approximately 1.2 million pediatric cancer survivors living in the U.S. and Europe alone [3, 4]. Yet, only 10% of children with cancer reside in HICs, and until recently, survival was strikingly low in LMICs [5, 6]. To address these disparate outcomes, the Global Initiative for Childhood Cancer (GICC) pledged substantial commitments towards achieving global survival of ≥ 60% by 2030. If successful, this initiative could result in a significant increase in the global number of childhood cancer survivors over the next few decades. Presently, effective survivorship care delivery is lacking in LMICs, highlighting the urgency to develop contextually appropriate strategies to meet the needs of this emerging population. New, multifaceted challenges arise with increased survival and ongoing care needs for cancer survivors due to higher risk of mortality and chronic health conditions as sequelae from disease or treatment [7, 8]. Survivors of pediatric cancer benefit from monitoring of recurrence or secondary malignancies, surveillance and management of physical and psychosocial effects, chronic disease medical care, health promotion and disease prevention [9, 10].

To improve follow-up care for the increasing number of survivors globally, it is important to understand how care is delivered and the barriers and facilitators, generally referred to as determinants, to caring for survivors. The Consolidated Framework for Implementation Research (CFIR) is a comprehensive framework used in implementation science to systematically evaluate multi-level determinants of evidence-based care [11, 12]. The CFIR provides a structured approach to identify and address key elements that impact the success of implementation by assessing factors across five characteristic domains: innovation, implementation process, outer setting, inner setting, and individuals. These domains have multiple constructs with definitions describing each [13]. After identifying barriers and facilitators, strategies for implementation can be examined. There are 73 evidence-based implementation strategies that were compiled by the Expert Recommendations for Implementing Change (ERIC) project. The CFIR–ERIC matching tool provides a way to select the most relevant evidence-based implementation strategies to overcome the identified barriers to implementing survivorship care [14, 15].

Existing research examines the current survivorship practices and challenges in HICs, including insurance status, disparities in care surrounding race and/or ethnicity, and lack of dedicated time or compensation for survivorship programs involving patient care and education [16, 17]. While barriers and facilitators to survivorship care have been examined in HICs, they have not been explored in LMICs. This study aims to review the literature to systematically examine existing evidence on barriers and facilitators to childhood cancer survivorship care in LMICs and identify appropriate implementation strategies that can be tested to improve survivorship care globally.

## Methods

### Data sources and searches

This systematic review was based on a larger systematic review that examined literature on global pediatric cancer survivorship outcomes (PROSPERO registration CRD42021242548) [18]. The parent review searched Global Index Medicus, CAB Direct, PubMed, and Embase to identify titles and abstracts that included studies published between 1980 and 2021 and reporting childhood cancer survivorship outcomes on patients ≤ 19 years of age at the time of cancer diagnosis with at least 5 years of follow-up. Some of the search terms included were “childhood cancer”, “survivor”, and “long-term effect” and a detailed list is available in the supplement of the initial study. The parent review extracted data from 1,558 articles in 43 countries and territories to examine primary and secondary outcomes of that study.

### Study selection

**Fig. 1:**
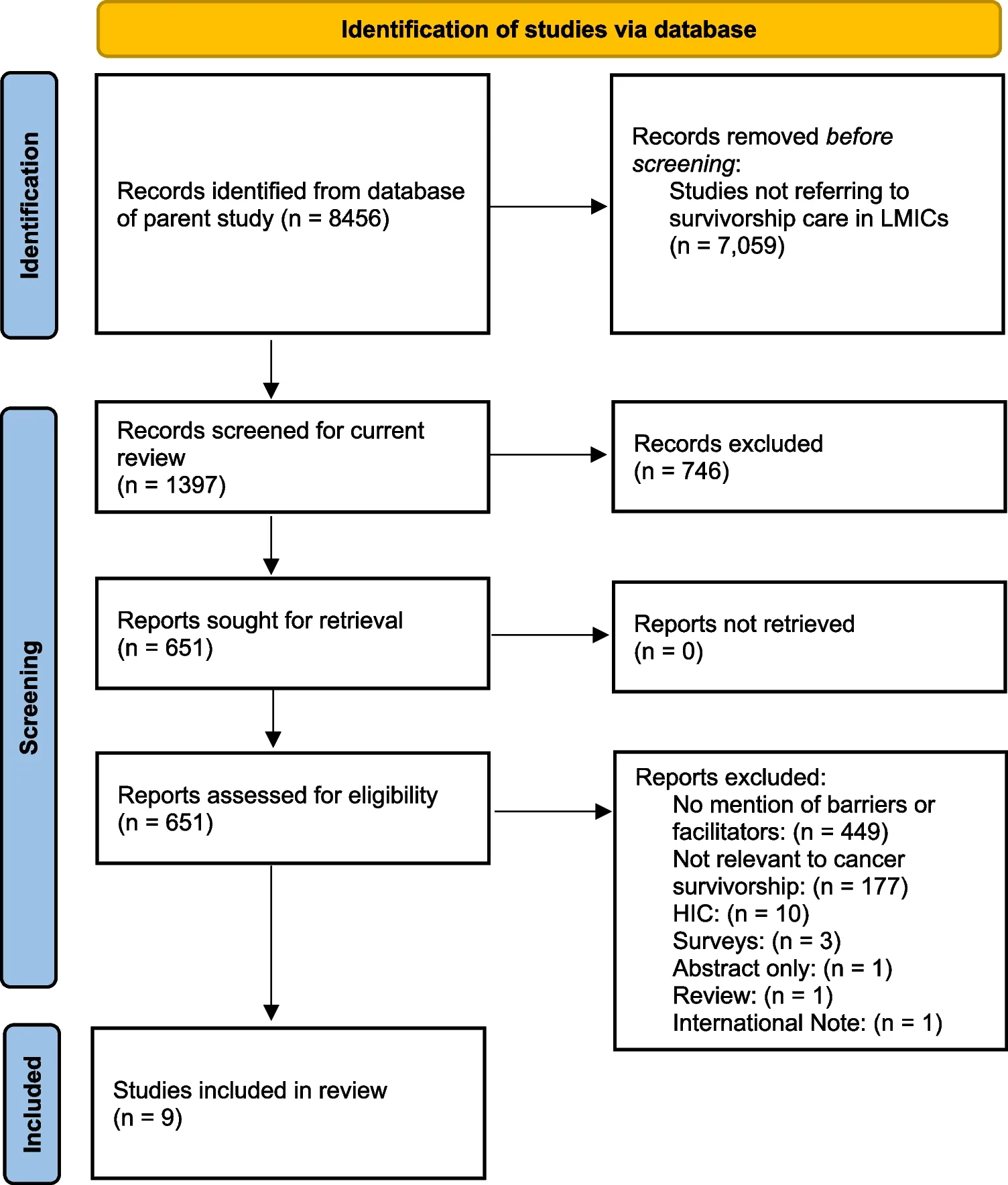
Preferred reporting items for systematic reviews and meta-analysis (PRISMA) diagram detailing the study selection process. LMIC = low- and middle-income country; HIC = high-income country

Using the initial 8,456 articles from the parent review, we completed a separate Preferred Reporting Items for Systematic reviews and Meta-Analyses (PRISMA) checklist to report this study, as there are no reporting guidelines specifically for secondary analyses of systematic reviews (Fig. 1). Inclusion criteria for studies were: 1) full-length, peer-reviewed research papers, 2) that were either observational or cross-sectional, 3) identified in the parent review as pertaining to LMICs, 4) published after 2000, and 5) that were focused on survivors of pediatric cancer, survivorship care (including development of survivorship clinics or programs), and evaluated determinants of survivorship care. Studies were excluded if they were: 1) not in English, Spanish, or Arabic, as these were languages of fluency for study team members, 2) reviews, essays, opinions, debates, protocol papers, trial registration papers, and conference abstracts, or 3) from HICs. The latter was stipulated since the interest of our study was LMICs, as defined by the World Bank.

Initial screening of titles and abstracts was conducted by a single reviewer (AS) on 1,397 articles, including 56 articles that were included in the parent study and within LMICs and 1,341 articles excluded from the parent study. The reviewer (AS) assessed the screened articles for inclusion. Of those, 651 articles originally were deemed potentially eligible for inclusion in this study. Two independent reviewers (GC and AS) then screened the titles and abstracts of these 651 articles. Disagreements regarding inclusion were resolved through consensus or by consultation with additional reviewers (MK, SM, and NG). Following this assessment, one additional article was included, resulting in a total of nine articles for data extraction.

### Data extraction and synthesis

Data were evaluated and extracted from the final nine articles (AS). A secondary reviewer (GC) examined the articles to augment or amend the extracted data. The two reviewers (AS and GC) then discussed discrepancies until reaching consensus. CFIR was used to guide the extraction of factors influencing how survivorship care is implemented in a global context.

Determinants were separately extracted into three domains: 1) the outer setting domain evaluated environmental implications to survivorship care, 2) the inner setting domain evaluated the organizational implications to care, and 3) the individuals domain evaluated individual-based implications to care. No specific barriers under the domains of innovation or implementation process were extracted as we did not focus on specific innovations in these settings. Additionally, we included general article information (i.e., year published, geographic location, study type) and strategies for implementing survivorship care. Data were summarized within each domain to generate a comprehensive understanding of barriers and facilitators to survivorship care in LMICs.

Barriers and facilitators identified from selected articles were mapped to constructs as detailed in the 2022 updated CFIR (Table 2) by one reviewer (NG) with expertise in implementation science and pediatric oncology, and disagreements were resolved by consensus discussion (SM, MK, NG) [12]. Using the conceptual linkages to CFIR barriers as established by the CFIR-ERIC matching tool, expert-opinion strategies were explored to address these barriers. One reviewer (NG) established the most appropriate connections between identified barriers and prioritized strategies with confirmation through consensus discussion (SM, MK, NG). Certain identified barriers did not have matched strategies (Fig. 3). As determined in Waltz et al. ERIC strategy rankings were denoted by color association with green referring to level 1 and yellow referring to level 2 endorsements [15]. Currently, facilitators are not examined in the CFIR-ERIC tool. Updated information regarding this tool is available at the CFIR guide website (cfirguide.org).

## Results

**Fig. 2:**
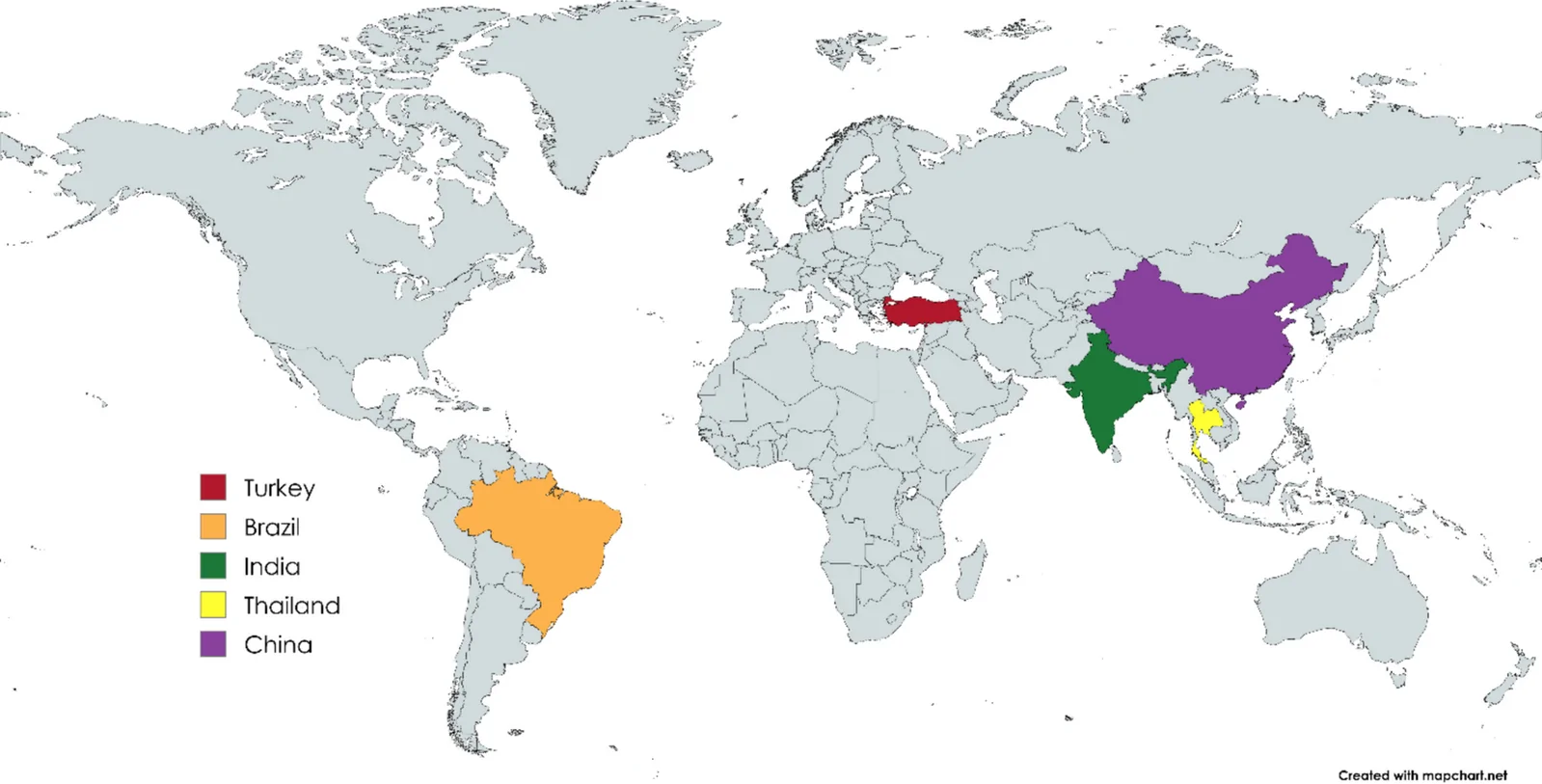
Countries from which included studies were published

A total of 8,456 articles were reviewed. The search identified nine eligible articles mentioning barriers and facilitators to survivorship care across five countries (Fig. 2). A majority of articles were excluded for not mentioning survivorship in LMICs (83.5%) Table 1.

**Table 1:** Characteristics of the included studies

Author	Year published	Years studied	Geographic location	Study design	Sampling size	Summary of barriers	Summary of facilitators
Rathore et al	2020	2017	India	Prospective	59 centers	• Insufficient resources and personnel • Lack of guidelines specific to region • Distance to clinics • Low health literacy surrounding survivorship care	None reported
Incesoy Özdemir et al	2020	2016–2017	Turkey	Cross-sectional	21 centers	• Insufficient resources and personnel • Lack of guidelines specific to region	• Existing clinic to support survivorship
Marinho et al	2020	1981–2015	Brazil	Retrospective	1 center	• Insufficient infrastructure • Political and social influences • Distance to clinics • Resource-limited medical systems	• Multidisciplinary team collaborating to provide care during encounters
Tonorezos et al	2018	2012	Brazil, India, and Turkey	Prospective	3 countries	• Lack of financial coverage for continuous patient care • Lack of guidelines specific to region • Distance to clinics • Low health literacy surrounding survivorship care • National policies impeding survivorship care	• Multidisciplinary team collaborating to provide care during encounters • Organizational support in building and sustaining survivorship clinics • Sufficient materials and personnel
Arora et al	2016	2013–2014	India	Prospective	42 centers	• Low health literacy surrounding survivorship care	None reported
Sun et al	2011	1984–2009	China	Retrospective	1 center	• Low health literacy surrounding survivorship care	• Existing clinic to support survivorship
Pakakasama et al	2010	2003–2005	Thailand	Cross-sectional	10 center	• Insufficient resources and personnel • Lack of guidelines specific to region • Difficult transition from pediatric to adult survivorship care	• Use of Children’s Oncology Group long-term follow-up guidelines
Kurkure et al	2003	1991–2002	India	Retrospective	1 center	• Lack of financial coverage for continuous patient care • Low health literacy surrounding survivorship care	• Multidisciplinary team collaborating to provide care during visits
Rigon et al	2003	1999–2000	Brazil	Retrospective	1 center	• Low health literacy surrounding survivorship care	None reported

### Barriers

**Fig. 3:**
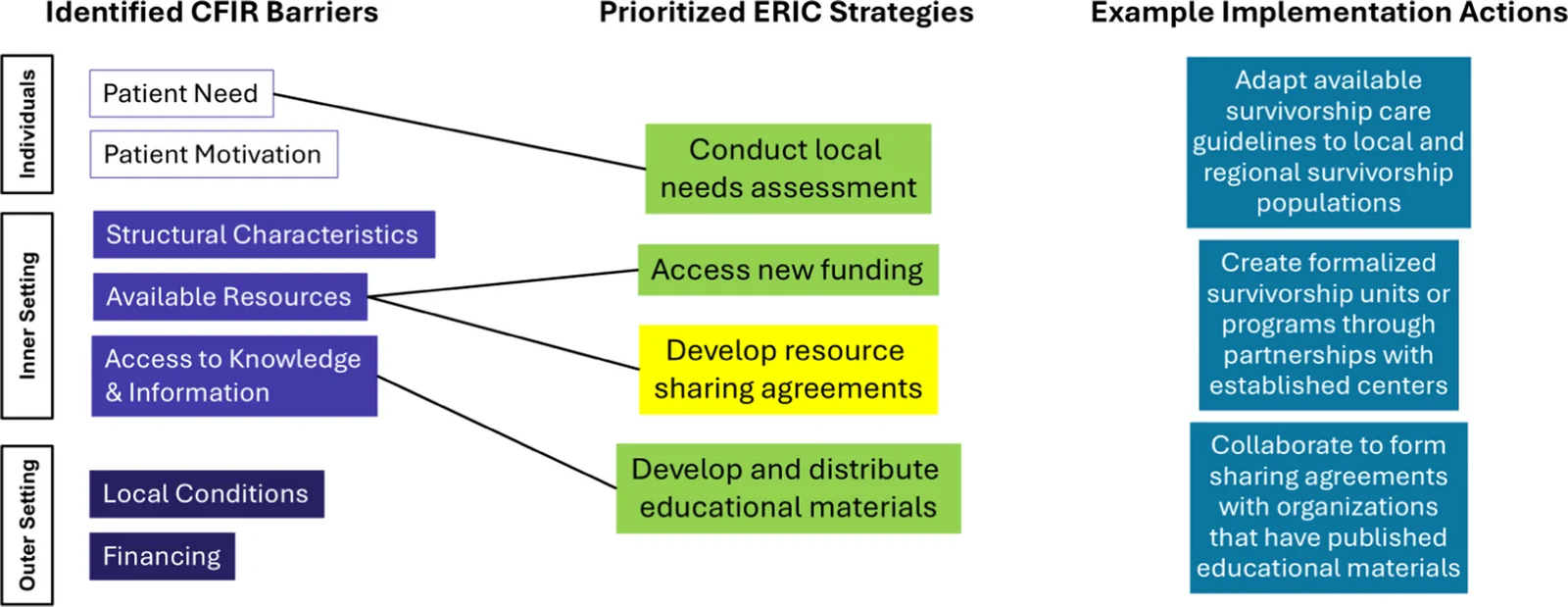
Roadmap for implementation of pediatric cancer survivorship care in LMICs. Lines represent a connection between the identified barrier and strategy in the CFIR-ERIC tool. A green box with connector represents a level 1 endorsement and a yellow box with connector represents a level 2 endorsement per the CFIR-ERIC tool. LMICs = low- and middle-income countries

We identified barriers pertaining to several CFIR constructs (Table 2, Fig. 3). Further descriptive analysis and details of these barriers are noted under their respective domains below.

**Table 2:** Common identified determinants and assigned 2022 CFIR constructs

Barrier	Construct
Health literacy	Patient need and motivation
Personnel scarcity	Structural characteristics
Limitation of funding, space and materials	Available resources
Minimal or no regional specific guidelines	Access to knowledge and information
National or regional policies for care	Local conditions
Government and/or private funding	Financing
Facilitator	
Multidisciplinary collaboration	Communications
Bilingual information sharing	Access to knowledge and information

#### Outer setting

Across all countries examined, there was a notable absence of dedicated survivorship care clinics, indicating a broader systemic issue. Additional barriers, particularly prominent in studies from India, included policy and governmental challenges. These were further described as a lack of state-funded social or economic support for cancer care, inadequate policies addressing medical care, employment, or health insurance for childhood cancer survivors, and restrictions on accessing health and life insurance for all cancer survivors [19–22].

In India, studies also highlighted deficiencies in health registries for monitoring childhood cancer survivors and insufficient oncology training and education [22]. Two studies assessing barriers in Brazil further emphasized the impact of social and political factors in impeding continued care for survivors [23, 24]. Furthermore, a lack of structured guidelines for survivorship care was identified as a significant challenge in Thailand, India, and Brazil [20].

#### Inner setting

Inner setting barriers, or organizational level barriers, varied across countries. A common challenge observed in all countries studied was a lack of clinical resources, encompassing constraints such as insufficient time, limited space, and understaffing [22, 25]. Moreover, studies conducted in India, Turkey, and Brazil highlighted the prevalence of hospital or clinic clusters in specific cities or regions, posing a significant obstacle for individuals from other parts of the country, rendering them isolated from accessing survivorship care services.

#### Individuals

At the individual level, a common thread across all mentioned countries was the prevalence of low health literacy among survivors, families, and healthcare providers [20, 22, 26]. Additionally, challenges such as long distances to the nearest clinic and lack of transportation were consistently cited as barriers to accessing care [22, 23].

In studies from India, families faced additional hurdles such as the significant time investment required to reach clinics, often conflicting with work or school commitments. Furthermore, there was perceived lower prioritization for survivorship care follow-up among patients, compounded by financial constraints that forced families to rely on their own resources for cancer care and follow-up, consequently hindering access to essential services [19].

### Facilitators

Facilitators were infrequently discussed and only identified within the inner setting domain of the CFIR. Across studies and countries, there were no common facilitators; however, there were some mentioned in specific contexts. For example, a study focusing on survivorship care in India highlighted the effectiveness of providing bilingual information on cancer treatments and side effects to accommodate the diverse linguistic landscape of the country [22].

A study from China examined late effects in childhood cancer survivors and identified the presence of established clinics for long-term follow-up as a key facilitator in ensuring patient adherence to care protocols [26]. Similarly, research conducted in Brazil emphasized the importance of optimizing interdisciplinary communication and collaboration among healthcare professionals, including the consolidation of interdisciplinary visits to minimize barriers to patient travel [23].

### Implementation strategies

Many strategies exist for implementation of a specific innovation. Formal expert recommendations have been established with the ERIC project and can be conceptually categorized in groups such as engaging consumers, evaluation, changing infrastructure, adapting to context, stakeholder relations, financial, supporting clinicians, training, and education [27]. Despite the critical role of implementation strategies in enhancing childhood cancer survivorship care, few studies explicitly addressed this. Studies based in India outlined several implementation initiatives:

1. Development of After Completion of Therapy clinics aimed at providing comprehensive medical, neuropsychological, and social evaluations for disease-free patients over a two-year post-therapy period [19].
2. Implementation of the CanKids-supported programs offered information, education, and communication materials focusing on fertility risks for childhood cancer survivors [21].
3. PICASSO initiative: Promotion and facilitation of hospital-based survivorship care clinics through collaborative efforts with the Indian Cancer Society [20].

Notably, a study conducted in Turkey highlighted plans to promote and publicize nationally structured childhood cancer survivorship guidelines, although implementation of this had not yet been realized [25].

Using the CFIR-ERIC matching tool, we prioritized strategies that would be relevant to the barriers identified (Fig. 3). To address resource limitations, accessing new funding was listed in the CFIR-ERIC tool as a level 1 strategy. Other strategies of level 1 ranking from the matching tool that targeted individual domain barriers included developing educational materials and local needs assessment. Many level 2 (lower) strategy recommendations were present in the analysis, with the most noteworthy being resource sharing agreements, which would effectively decrease innovation costs and reduce the need to access new funding.

## Discussion

Utilizing the CFIR framework, we identified key barriers and facilitators to childhood cancer survivorship care delivery in LMICs. Only nine studies addressed these factors in our review. Frequently observed findings included resource limitations within clinical care settings and institutions. While necessary, accessing new funding to overcome this barrier can be difficult, especially in LMIC settings. Thus, we propose other strategies such as resource sharing agreements and educational material adaptation to local needs. Further exploration of barriers and facilitators to support specific innovations such as clinical decision support tools from context-based guidelines or application of survivorship care models to existing infrastructure is needed.

Increasing investments seeking to improve global cure rates for childhood cancer are anticipated to result in an emerging population of childhood cancer survivors in LMICs, emphasizing the importance of our findings in addressing the health care needs of this vulnerable population. A recent publication has looked at strategies for promoting equity within survivorship care in limited resource settings using the World Health Organization (WHO) GICC Cure*All* framework and opinion among international survivorship experts, which can be a valuable resource for clinicians in LMICs [28]. Our systematic analysis of the reported barriers in the literature and identification of evidence-based implementation strategies that specifically overcome those barriers further strengthen the proposed approaches in the aforementioned publication. These strategies can then be incorporated to support the implementation of specific innovations such as survivorship care guidelines and reference resources for patients and clinicians.

Current projects are underway to engage clinicians in LMICs in generating context-tailored guidelines through the Adapted Resource and Implementation Application (ARIA) Guide and international collaborative guidelines through the International Guideline Harmonization Group (IGHG) [29, 30]. The ARIA Guide is a web-based tool under development through a collaboration between St. Jude Children’s Research Hospital and the International Society of Paediatric Oncology (SIOP) that will disseminate resource-adapted guidelines that are feasible to implement in LMICs, as well as educational materials and interactive tools to support care delivery for clinicians. By contextually adapting existing, gold standard survivorship guidelines (e.g., those of the IGHG) for local context, this effort is anticipated to dramatically expand access and enhance implementation of recommendations within real-world settings. This is an example of a targeted innovation that can then be implemented broadly to meet the needs of LMICs in delivering evidence-based survivorship care.

Childhood cancer survivorship care involves a comprehensive approach to addressing the physical, psychosocial, and educational needs of survivors, with the aim of optimizing their long-term health and quality of life. This broad approach of care poses a significant implementation challenge. Regardless, childhood cancer survivorship care is essential in the prevention, surveillance, and intervention of effects that can stem from cancer treatment, even years to decades after [31]. By empowering patients and providers in a variety of global contexts, equitable delivery of childhood cancer survivorship care is achievable. Multiple components of care necessitate coordination, often among different providers and health care systems. There are several models of survivorship care that strive to achieve optimal care coordination, including oncology-led, primary care-led, shared care, multidisciplinary clinics, and risk-stratified care. Models of care ultimately refer to the type of care being delivered and which providers are delivering the care, when and to whom, and these models differ substantially within and across countries [20, 32]. Certain care models were described as facilitators in our study, including programs that prioritized collaboration and multidisciplinary visits to reduce travel burden for patients.

This review has various limitations. Firstly, only nine articles met inclusion mentioning determinants to survivorship care in LMICs, and the quality of these manuscripts was not systematically assessed. All the studies included had predated the COVID-19 pandemic, which could have subsequently resulted in noteworthy changes in barriers and facilitators. Changes in health care delivery, such as telehealth, may have changed survivorship care access and rendered some of the barriers less relevant. Additionally, determinants detailed in the articles were from the perspective of providers and researchers. Although individual domain barriers could be extracted from the articles, these lacked the perspectives of patients, families, and caregivers, signaling an important gap to overcome in future studies.

In summary, limited published data exist regarding determinants of childhood cancer survivorship care delivery in LMICs, with low resource availability being a frequently reported barrier in the articles identified. As accessing funding can be difficult, resource sharing of contextually adapted guidelines and educational materials can serve as a strategy that aligns well with the WHO GICC Cure*All* enabler of leveraged financing. This will help to catalyze implementation efforts to improve survivorship care in LMICs; however, further work is needed to better understand determinants in a variety of global contexts.

## Data Availability

No datasets were generated or analysed during the current study.
